# Adsorption Characteristics of Oxytetracycline by Different Fractions of the Organic Matter from Humus Soil: Insight from Internal Structure and Composition

**DOI:** 10.3390/ijerph17030914

**Published:** 2020-02-01

**Authors:** Mengya Luo, Shengke Yang, Siqi Shen, Yu Li

**Affiliations:** Key Laboratory of Subsurface Hydrology and Ecological Effects in Arid Region (Chang’an University), Ministry of Education, School of Environmental Science and Engineering, Chang’an University, Xi’an 710054, China; 2018229049@chd.edu.cn (M.L.); 2018229032@chd.edu.cn (S.S.); 2018229051@chd.edu.cn (Y.L.)

**Keywords:** Humus, organic matter fractions, oxytetracycline, adsorption

## Abstract

For minimizing the transport of antibiotics to groundwater, the migration of antibiotics in soils should be investigated. Soil organic matter can affect the migration of antibiotics. To date, the influence of aromatics and aliphatic content of organic matter on the adsorption of antibiotics has been controversial. To better understand the reaction mechanism of soil organic matter with antibiotics, this study investigated the adsorption of oxytetracycline (OTC) by humus soils (HOS) and their fractions. HOS were sequentially fractionated into four organic fractions, including the removal of dissolved organic matter (HRDOM), removal of minerals (HRM), removal of free fat (HRLF), and nonhydrolyzable organic carbon (HNHC). Moreover, batch experiments revealed that adsorption capacity was ordered by HNHC > HOS > HRDOM > HRLF > HRM. SEM images and N_2_ adsorption/desorption isotherms indicate that adsorption capacity is independent of the external structure. However, adsorption capacity is related to the internal structure and composition. Combination analysis with elemental composition and infrared spectroscopy showed that the adsorption capacity of HRM, HRLF, and HNHC had a good positive correlation with aromaticity, but a negative correlation with polarity and hydrophilicity. Additionally, the rule of binding affinity between OTC and functional groups with different properties was summarized as aromatic > polarity > hydrophilic.

## 1. Introduction

Antibiotics are not only used in human medicine for treatment, but also widely used in livestock feed in recent years [1]. Antibiotics have been extensively detected in soil, overland runoff, underground water, and sediment [2]. Oxytetracycline (OTC), belonging to the tetracyclines class of antibiotics, is used in the livestock and poultry industry worldwide owing to its high quality and low price [3]. Since solid retention time is commonly one order of magnitude higher than hydraulic retention time, certain antibiotics and degradation products might accumulate in the soil, leading to higher soil concentrations than in raw sewage [4,5,6]. It was reported that OTC were detected most frequently in farmland soil of Canada and Britain, at concentrations as high as 513 μg/kg and 1691 μg/kg [6,7] respectively. The concentration of OTC in the sediments around an aquaculture plant in China was as high as 300 mg/kg [8]. Antibiotics can not only damage the soil environment, but also affect human health [9,10,11,12]. More importantly, antibiotics are moved with soil water to enter the underground water and cause pollution. Thus, understanding the migration of antibiotics in soil has significance for the protection of groundwater.

The fate of antibiotics in soil are mainly controlled by the adsorption/desorption function of soil. The adsorption behavior of antibiotics is further affected by clay minerals, metal oxides, and organic matter in soil [13,14,15]. Although there is little organic matter in soil, it can strongly affect the fate of antibiotics in soil [15,16]. Soil is negatively charged owing to organic matter surface contained polar functional groups, for instance, hydroxyl, carbonyl, and carboxyl. The polar functional groups of antibiotics can be adsorbed to the surface of the soil through electrostatic interaction [17], hydrogen bond [18], synergistic interaction, and complexation mechanism [19]. The influences of organic matter have been extensively reported during the adsorption/desorption process of antibiotics in soil. Many have found that there is a simple positive relationship between the content of soil organic matter and adsorption capacity [20,21]. However, Pils et al. [22] found that some soil organic matter could cover up adsorption points, leading to reduce the absorption of antibiotics in the soil. Therefore, such a simple positive correlation denotes a lack of rationality. In summary, the previous studies suggested that organic matter could affect the migration of antibiotics in soil. However, there remains controversy about the influence of organic matter on antibiotic adsorption at present. Further studies on accurate feature and mechanism of soil organic matter adsorption/desorption for antibiotics are necessary.

According to solubility, Weber et al. [23] separated soil organic matter operationally into fulvic acid (FA), humic acid (HA), and humin (HM). The adsorption capacity of hydrophobic organic pollutants are order: FA < HA < HM [24]. However, the affinity of polycyclic aromatic hydrocarbons for HM is lower than that for HA [25]. Sun et al. [26] found that nonhydrolyzable organic carbon (NHC) was separated into kerogen graphitic (KC) and black carbon (BC). The adsorption capacity of Phenanthrene (Phen) by different fractions of organic matter are: NHC≈BC>KC. Ran et al. [27] further found the adsorption capacity of Phen by NHC fraction is negative with respect to aromatic carbon content. However, Jin et al. [28] found aromatic domains of NHCs acted as p-acceptors to promote the adsorption of Phen. Obviously, for hydrophobic organic pollutants only, the adsorption mechanism is complex. OTC as hydrophilic organics pollutants contain polar functional groups (e.g., -OH -CONH and -NH(CH_3_)_2_), which make the adsorption process more complex than hydrophobic organic pollutants, but must have its specific regular. It is necessary to explore different types of regular pollutant adsorption by different fractions of organic matter. Besides the property of antibiotics and the content of soil organic matter, the source, fractions, and functional groups of organic matter can affect adsorption. For understanding the reaction and mechanisms between organic compounds and different fractions of matter organic, the roles of composition and internal fine structure in their adsorption should be further investigated.

It should be emphasized that the composition and structure of organic matter differs greatly depending on the soil type [29]. Humus soil (HOS) is a kind of geologically young soil, formed by dead branches and leaves after long term decay fermentation. Compared to the sedimentary and peat soils studied previously, the of HOS structure is loose and porous and contains carboxyl, hydroxyl, and some other functional groups, with a more representative and typical adsorption of pollutants [30,31]. Therefore, the purpose of this study was as follows: (1) HOS was sequentially divided into the removal of dissolved organic matter (HRDOM), removal of minerals (HRM), removal of free fat (HRLF), and nonhydrolyzable organic carbon (HNHC) to evaluate different fractions adsorption characteristics and mechanisms on OTC. (2) Compare and analyze the adsorption relationship of OTC among different dissolve organic matter (DOM) coming from different soils. (3) Reveal the effect of functional groups of different fractions on OTC adsorption.

## 2. Materials and Methods

### 2.1. Extraction Treatment of Sample

**Figure 1:**
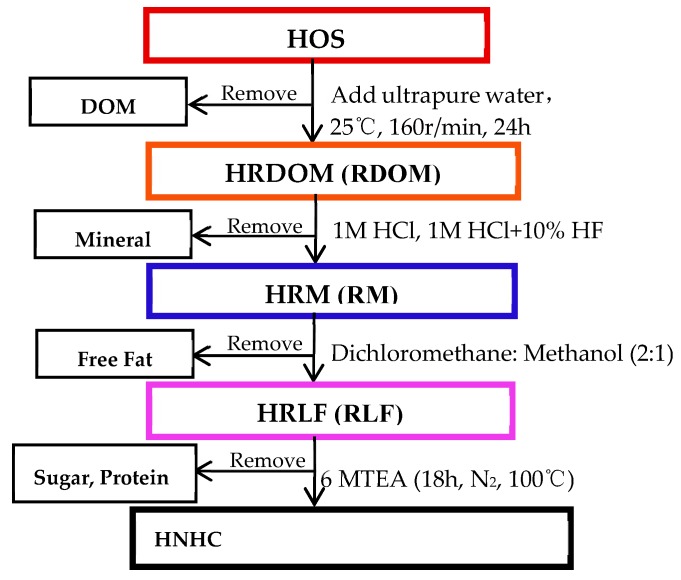
Extraction treatment of the organic matter of humus soil.

The HOS (original sample) was collected from the 5–15 cm below the ground under the surface of the shrub along the Weihe river basin (34° 23′ 41.86″ N, 108° 52′ 38.99″ E). After removing the impurities, the HOS was naturally dried in the dark. Then was pestle through a sieve (80 mesh). It was sealed and saved at 4 °C before used (all the samples were detected without OTC). Figure 1 shows brief steps [31,32,33].

The first step was to obtain HRDOM fraction: 30 g of HOS and 300 ml of ultrapure water were shaken on a shaker for 24 h in an Erlenmeyer flask, and then the solution was centrifuged. The supernatant was soil dissolved organic matter (DOM), which was saved in the dark at 4 °C after filtering through a 0.45 μm filter. The centrifuged solid was HRDOM fractions after drying. 

The second step was to obtain HRM fraction: Firstly, the HRDOM fraction was dissolved in 1 M HCl, shaken for one day, and centrifuged for 30 minutes to remove carbonate. To remove the silicate, the solid after centrifuge was shaken with a 10% mixture of 1m HCl and HF for 4 days. Finally, the solid was HRM fraction after centrifuging again and drying. 

The third step was to obtain HRLF fraction: The HRM fraction was extracted under ultrasonic conditions for 15 minutes, and the supernatant was removed by centrifugation. After repeated 5 times, the solid portion obtained was the HRLF fraction. The extract solution was consisted of dichloromethane and methanol (2:1, V/V).

The fourth step was to obtain HNHC fraction: The HRLF fraction was successively hydrolyzed with 2M trifluoroacetic acid, 4M and 6MTFA and 6M HCl in Teflon furnace at 100 ° C for one day. Then, the supernatant was removed after centrifugation. The solids are then washed with ultrapure water to neutrality, which is the HNHC fraction. Fill with nitrogen for not less than 5 minutes before each hydrolyze. 

All shaken parameters were 160 rpm for 24 h and centrifugation parameters were 6000 rpm for 30 min. All sample were dried at 50 °C before use. Table 1 shows the total organic carbon (TOC) content and pH values.

### 2.2. Batch Experiment

The batch experiments followed the Organization for Economic Cooperation and Development method [34]. Under neutral conditions, 0.05 g of different fractions and 5 mL of 10.0 mg/L OTC solution at 30 °C which shaking at 160 rpm for one day in the dark. The kinetic data were obtained at 2, 4, 8, 12, 20, 24, 28, 36 and 48 h. For adsorption isotherm and thermodynamic experiments, OTC initial concentrations were between 5 mg/L and 25 mg/L, the OTC solution temperatures were 20 °C, 30 °C, and 40 °C, respectively. The samples were filtered through a 0.45-μm filter before ultra-performance liquid chromatography (UPLC). All of the experiments were performed in triplicates. 

Batch experiments were performed in the pH range of 2–10 for explore the influence of pH on OTC adsorption. To explore the influence of DOM types on OTC adsorption, 1 mL exogenous DOM from decay plant (PDOM) and chicken manure (MDOM) were added into the removal of DOM of humus and sediment soil for batch adsorption experiments. PDOM and MDOM extraction methods were consistent with humus soil.

### 2.3. Sample Analysis

OTC in solution was detected by UPLC. The chromatographic column was ACQUITY UPLC BEH C18 column (2.1 × 150 mm, 1.7 μm particles). The column temperature was 40 °C. Injection volume was 5 μL and flow rate was 0.2 mL/min. Mobile phase composition was methanol: water = 60:40 (v/v). The equations of adsorption models of kinetics and isotherm were employed to fit the experimental data, as shown in Table 2 [35,36].

### 2.4. Characterization of Different Fractions

Quanta 200 scanning electron microscopy was used to observe the morphology of different fractions. The pore-size distributions and specific surface areas were analyzed volumetric system (ASAP 2460, Micromeritics, America) through the nitrogen adsorption/desorption isotherms. Vario ELⅢ elemental analyzer was used analysis elemental (C, H, N). The fluorescence spectra (F-7000; Hitachi, Japan) of DOM were recorded. The excitation and emission wavelengths (Ex and Em) ranged from 220 to 600 nm and 250 to 600 nm, respectively. The scan rate was 1200 nm/min. The different fractions were characterized by Tensor 27 infrared spectroscopy. The Elementar Vario TOC was analyzed TOC.

### 2.5. Quality Assurance and Control

The calibration curve was prepared by more than seven standard solutions in the linear range. The linear range of OTC test was 1–100 mg/L. The detection limits of quantification were 0.051–0.057 mg/L, while relative deviations were 2.6%–10.8%. Recoveries of the OTC were determined in the range of 79% and 113%. OTC were not detected in the blank sample, so the data were reliable.

## 3. Results and Discussion

### 3.1. Adsorption Studies

#### 3.1.1. Adsorption Kinetic

**Figure 2:**
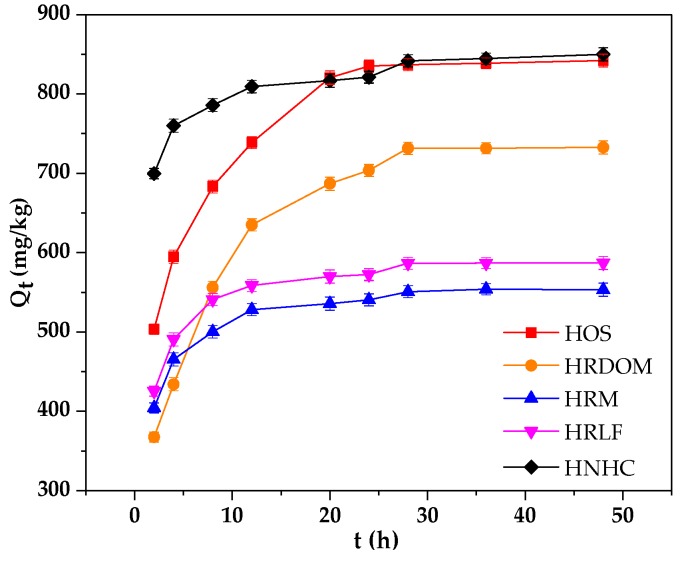
Adsorption characteristics of OTC by different fractions.

As shown in Figure 2, the adsorption rate of HRM, HRLF, and HNHC were significantly higher than that of HOS and HRDOM. This may be due to the presence of DOM and inorganic minerals so that further adsorption requires greater activation energy, and the adsorption rate becomes slow. However, HRM, HRLF, and HNHC are removed of minerals, lipids, carbohydrates, and other pollutants which can quickly reach the adsorption equilibrium. The specific amounts of five fractions were calculated as the equation listed below:

(1) $$q_{t}=\left(\frac{C_{0}-C_{t}}{m}\right)V$$

where *q*_*t*_ (mg/kg) is the quantity of OTC adsorbing onto different fractions at a predetermined time, *C*_*t*_ (mg/L) is the instantaneous concentration of OTC in solution at time t (h).

The corresponding fitting parameters are listed in Table 3. The smaller the residual sum of squares (RSS/dof), the better the model fits. Hence, the equilibrium adsorption capacity (Qe, cal) of the pseudo-second-order kinetics model is close to the actual equilibrium adsorption capacity (Qe, exp). The K values of adsorption by HRM, HRLF, and HNHC are significantly larger than those of HOS and HRDOM, which may be related to the microstructure of different fractions.

#### 3.1.2. Adsorption Isotherms

**Figure 3:**
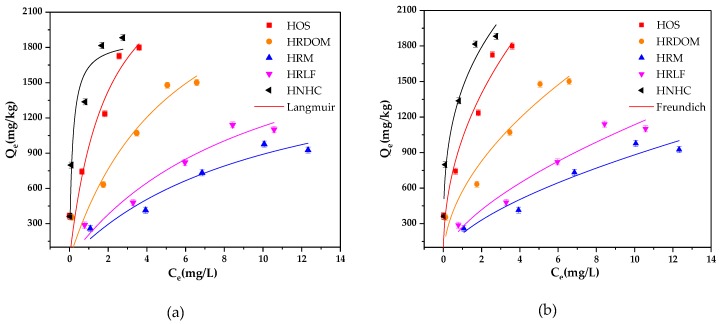
The fitted adsorption isotherms of OTC by different fractions: (**a**) Langmuir model; (**b**) Freundlich model.

The non-linear fitting of Langmuir and Freundlich model to isothermal adsorption data is shown in Figure 3 and Table 4. All samples are fitted well with the Freundlich model by considering lower Akaike’s information criterion (AICc) [37]. The degree of nonlinearity and difficulty of adsorption both have negative correlations with the value of 1/n. The values of 1/n range from 0.37 to 0.63, which indicate an obvious non-linearity process for the OTC adsorption by different fractions in humus soil. HNHC is speculated to be the most likely component for OTC adsorption, followed by HOS, HRDOM, HRLF, and HRM. In addition, generally, the adsorption capacity has a positive value of K_F_ [38]. Therefore, the adsorption capacity of each fraction is ordered: HNHC > HOS > HRDOM > HRLF > HRM.

#### 3.1.3. Adsorption Thermodynamic

The thermodynamic characteristics of OTC adsorption can help to further understand the trend and extent of OTC adsorption by different fractions. The thermodynamic parameters can be calculated as following equations:

(2) $$\Delta {G=-RTlnK}_{0},$$

(3) $${lnK}_{0}=-\frac{\Delta H}{RT}+\frac{\Delta S}{R}$$

where Δ*G*, Δ*H* and Δ*S* are standard free energy change (kJ/mol), standard enthalpy change (kJ/mol), and standard entropy changes (J/mol k), respectively. *R* is the universal gas constant (8.314 J/mol k) and *T* is the temperature (°C). *K*_0_ is the thermodynamic equilibrium constant. The parameters are listed in Table 5. The values of Δ*G* are range from –9.67 to –22.33 kJ / means that the adsorption of OTC is spontaneous by different fractions at different temperatures. The Δ*G* values are increased with removal of DOM. The presence of DOM can prevent macromolecules from occupying adsorption sites on the soil surface. In addition, studies have shown the value of ΔG is –20~0 kJ/mol meaning physical adsorption. The chemical adsorption is –80~–400 kJ/mol [39]. However, the value of Δ*H* between 12.98 to 99.26 kJ/mol suggests that the adsorption of OTC by different fractions is endothermic. Increasing the temperature can promote adsorption. More heat is needed for chemical adsorption than physical adsorption, so the adsorption of OTC by different fractions is not simple physical adsorption. The value of Δ*H* of HRDOM, HRM, and HRLF are lower than HOS and HNHC, which implies that there is weak bonding between the OTC and HRDOM, HRM and HRLF. The positive Δ*S* value indicates that the adsorption is an entropy increasing reaction, which means it is likely that the adsorption of OTC may occur in the different fractions of an internal structure [40].

#### 3.1.4. Comparative Study

Compared with the sedimentary soil previously studied by our team was found that the adsorption capacity of different source of organic matter were different (Table 6). OTC adsorption capacity of humus soil was much larger than sedimentary soil [31]. This is verified by lower 1/n values and more negative Δ*G*. It was also found the adsorption capacity of humus soil to OTC was reduced, and the adsorption capacity of sediment soil was increased after removing endogenous DOM. The reason for this may be that different types soil have different functional groups.

### 3.2. Effects of External Factors

#### 3.2.1. Effect of pH on Adsorption

**Figure 4:**
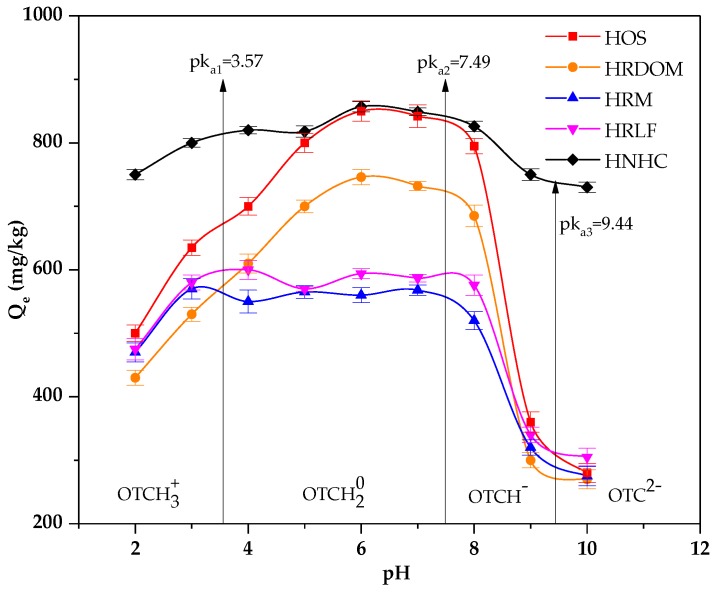
The effect of pH on the adsorption of OTC by different fractions.

From Figure 4, we found the pH conditions can affect the adsorption of fractions other than HNHC. In general, OTC is amphoteric so that OTC exhibits different electrical properties at different PH values [41,42]. Furthermore, when the pH > pHzpc, the surface of the soil is negatively charged. On the contrary, the surface of soil surfaces is positively charged [43,44]. The pHzpc of different fractions was 5.12~5.65. When the pH is under 3.00, the dimethyl ammonium group was protonated and exists as an OTCH_3_^+^. The different fractions are positively charged. The decreases of adsorption capacity since the rejection of the charge reduces the contact of the OTC with different fractions. Similarly, when the pH is above 7.00, OTC exists as OTCH^−^ or OTC^2−^ owing to the loss of protons and different fractions being negatively charged. Therefore, the adsorption capacity decreases. Between pH 3.3 and 7.7, because of phenolic diketone moiety loss of proton, it exists as OTCH_2_^0^. DOM can affect the adsorption of OTC through hydrophobic partitioning, complexation, and hydrogen bonding. Hence, the effects of PH on HOS and HRDOM are consistent [45]. It has been known that the zero charge point of most aluminosilicate minerals is around pH 3.00 [46]. Therefore, when the pH is lower than 3, there is no longer electrostatic repulsion between HRM and OTC. Fatty acid, polysaccharides, and protein have lots of oxygenous functional groups, which can compete for adsorption sites [47,48]. Therefore, the adsorption amount of HRLF for OTC is higher than HRM.

#### 3.2.2. Effect of DOM on Adsorption

**Figure 5:**
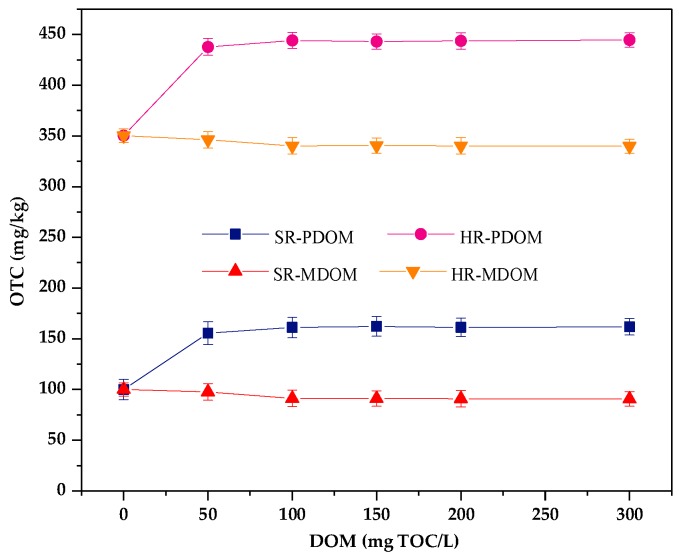
Effects of different DOM types on the adsorption of OTC in humus (HR) and sediment (SR) soil.

Endogenous DOM in different types of soil has opposite effects on the adsorption of OTC. Adding two types of exogenous DOM into removal of endogenous DOM of humus (HR) and sedimentary soil (SR). The results are shown in Figure 5. It was reported that PDOM (from decay plant) mainly contains humic-like substances and MDOM (from chicken manure) mainly contains protein-like substances [49]. It can be seen that the presence of PDOM enhances the adsorption of soil to OTC in all concentrations. However, the adsorption capacity of OTC by two soils is decreased with the increasing MDOM concentration. 

**Figure 6:**
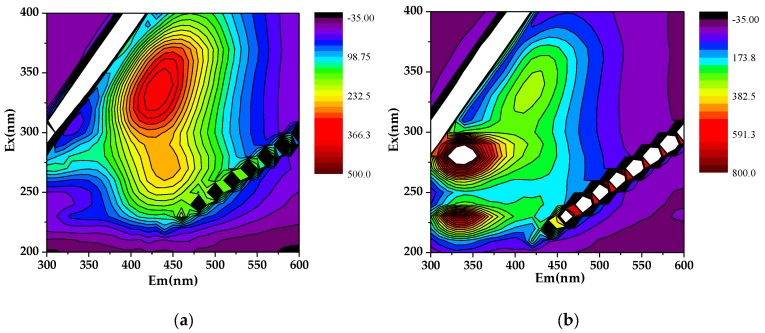
The 3D fluorescence spectra of DOM: (**a**) humus soil; (**b**) sedimentary soil.

The location and meaning of fluorescence peaks are investigated by many studies (Figure 6) [50,51,52,53,54,55]. Different peaks of endogenous DOM extracted from humus and sedimentary soil can be identified by three-dimensional fluorescence spectra. The endogenous DOM of humus soil (Figure 6a) has a high strength H3 peak at Ex/Em = 340/430, which is assigned to a lot of humic-like substances. Inversely, the endogenous DOM of sedimentary soil (Figure 6b) has two P2 peaks atEx/Em = 280/320 and a high strength peak at Ex/Em = 230/320, which are assigned to a lot of protein-like substances. Hence, the DOM of humus soli can promote the adsorption of OTC, while sedimentary soil does the opposite.

### 3.3. The Role of Internal Structure and Composition

#### 3.3.1. Morphology and Specific Surface Area Characteristics

**Figure 7:**
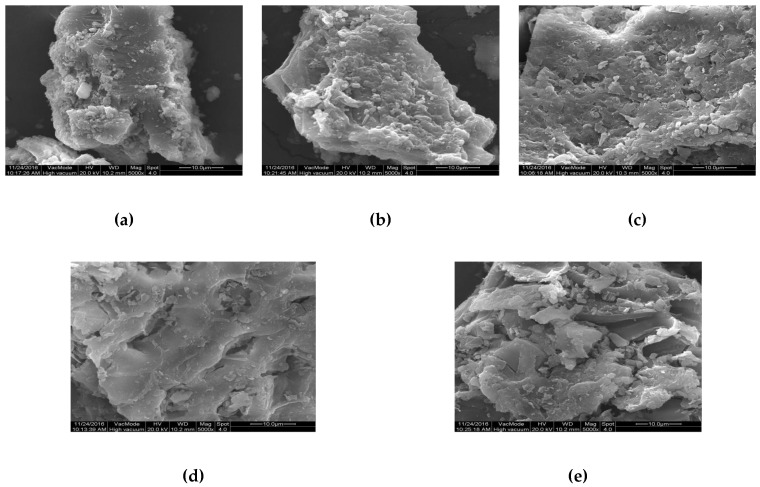
The micro-structure of: (**a**) HOS; (**b**) HRDOM; (**c**) HRM; (**d**) HRLF; (**e**) HNHC.

**Figure 8:**
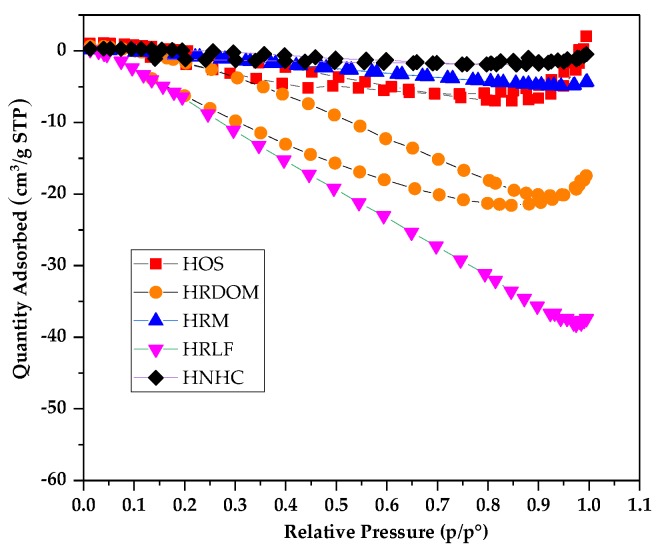
N_2_ adsorption-desorption isotherms of different fractions.

The micro-structure of humus soil in different fractions of organic matter was recognized using SEM. Figure 7 shows the micro-structure features of HOS, HRDOM, HRLF, and HNHC with 10μm under 5000 times. It can be seen that HRDOM have a rougher surface and more microporous structure than HOS, while adsorption capacity is reduced. Although HRM have more pore structures generated after the removal of minerals, the adsorption capacity is the lower than other fractions. The number of pores of HRLF decreases, but the adsorption capacity increases, as opposed to HRM, which may be caused by more adsorption sites after the removal of free fat. The pore structure of HNHC almost disappeared and the surface is smooth, but the adsorption amount is much larger than other fractions. With the sequential removal of DOM, minerals, and free fat, more effective adsorption domains in HNHC were exposed, so the adsorption sites blocked or occupied become accessible for the adsorption of OTC. These phenomena indicated that the adsorption capacity of different fractions to OTC is not completely dependent on the rough surface and microporous structure. In order to further verify this conclusion, the different fractions of organic matter were determined by BET (Table 7, Figure 8).

The organic matter of each fraction of the humus soil has a very smaller specific surface area than porous materials which have strong adsorption capacity. Among them, HRDOM, HRM, and HRLF are all undetected micropore volumes, while HOS and HNHC only have very small micropore volume. From Figure 8, the adsorption capacity of N_2_ by each fraction showed a downward trend as a whole. This is because the interaction between adsorbate molecules is stronger than between the adsorbate and adsorbent. It is further proven that a specific surface area of different fractions of organic matter are too small to purely physically adsorb. Except for HOS and HNHC showing a small amount of adsorption capacity under high relative pressure (P/P^0^), the remaining fractions have hardly the ability to physically adsorb OTC. It follows that the adsorption capacity is independent on the external structure. The adsorption capacity of different fractions of organic matter to OTC depends more on surface specific functional groups than surface area.

#### 3.3.2. Adsorption Site Distribution Characteristic

**Figure 9:**
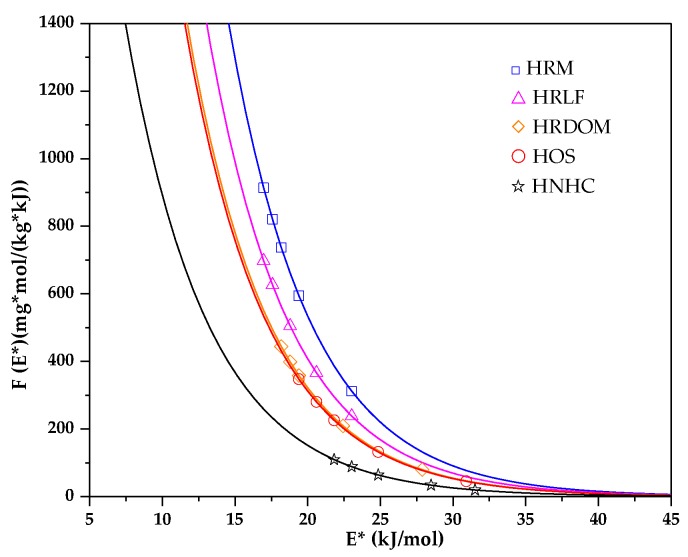
Site energy distribution based on adsorption isothermal model.

The theory of site energy distribution can provide an adsorption amount of the adsorbent surface site and the corresponding fractional function [56]. The humus soil in various states based on Freundlich model is shown in Figure 9. According to the solubility of OTC, the relative adsorption energy E* values of OTC in the experimental concentration range are in the range of 16.758–31.851 kJ·mol^−1^.

From Figure 9 it can be seen that the site energy distribution function decays rapidly by an exponential function. The adsorption sites of OTC in the HNHC fraction are distributed in the high energy region evenly, while the HRM and HRLF fractions are distributed in the low energy region evenly. In HOS and HRDOM fractions, the adsorption sites of OTC are distributed with a poor uniformity. The high energy adsorption sites of different fractions are covered by OTC in the low concentration range, thereby increasing the uniformity of surface organic matter. When adsorbed in a high concentration range, the organic matter of each fraction has a strong adsorption capacity for OTC, and there exist few high-energy adsorption sites. The adsorption process mainly occurs in the low energy region. Within a certain range, the area under the site energy distribution curve can be interpreted as the maximum number of sites available for adsorption. Therefore, OTC has more adsorption sites in the HNHC fraction, followed by HOS, HRDOM, HRLF, HRM. This indicates that the adsorption capacity of each fraction is ordered as: HNHC > HOS > HRDOM > HRLF > HRM. This result is in good accordance with the obtained data from the isothermal adsorption model.

#### 3.3.3. The Role of Functional Groups

**Figure 10:**
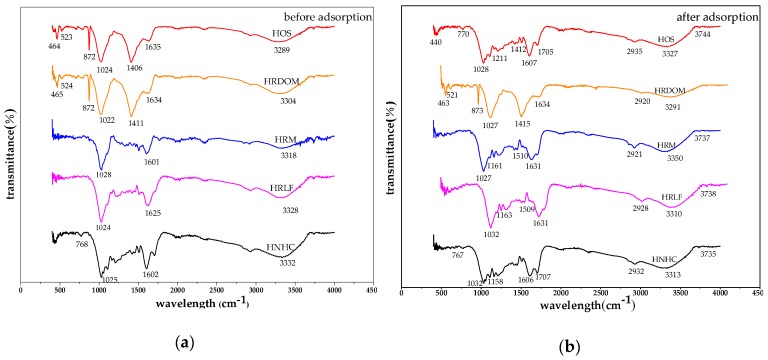
The infrared spectra of different fractions: (**a)** before adsorption; (**b)** after adsorption.

Before and after the adsorption of OTC, the changes of infrared spectra are shown in Figure 10. (1) The Si-O-Si stretching vibration peak of 872 cm^−1^ disappears or decreases in HOS and HRDOM samples, and the bending vibration peaks of Si-O in the vicinity of 790 cm^−1^, 520 cm^−1^, and 460 cm^−1^ show displacement in different degrees, which indicates that the complexation or coordination may have occurred, and the interaction intensity of HOS and HRDOM are different; (2) For all fractions, the C-O stretching vibration absorption peak is near 1020 cm^−1^ and the C=C telescopic vibration peak located at 1600–1635 cm^−1^, while the shifts and vibration intensity also changed in different degrees. In other words, the charge transfer, coordination, or complexation with different effects are produced; (3) The O-H bending vibration peaks of HOS and HRDOM around 1400 cm^−1^ almost disappear in HOS and weakened in HRDOM, indicating that hydrogen bonding is occurred; (4) Owing to the association between the -OH molecules, which are named hydrogen bonds, the -OH near 3300 cm^−1^ stretching vibration absorption peak shifts downward and the peak shape of varied degrees of changes. The peak shape of HRM, HRLF, and HNHC become sharp significantly, showing that the intensity of hydrogen bonding between the fractions and OTC is different. Further, after OTC adsorption at each fraction, the peak intensity of the 400–1600 cm^−1^ region also changes, which further indicates the interaction with OTC molecules varied.

The atomic ratio about H/C, (N+O)/C and O/C indicate the aromatics, polarity, and hydrophilicity of different fractions, respectively (Table 8).

**Figure 11:**
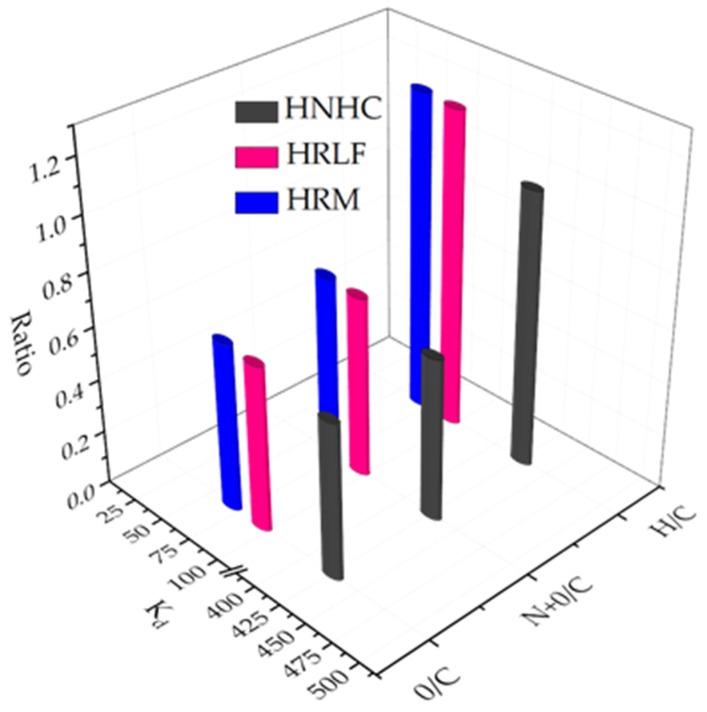
Relationship between adsorption coefficient K_d_ of HRM, HRLF and HNHC and H/C, (N+O)/C, O/C.

According to Figure 11 and Table 8, It can be seen that aromaticity, polarity and hydrophilicity all effect the adsorption capacity. For the fractions of HRM, HRLF, and HNHC based organic matter, the adsorption coefficient K_d_ has a good positive correlation with aromaticity, while it has a negative correlation with polarity and hydrophilicity. HNHC has the largest adsorption capacity because of its highest aromaticity and lowest hydrophilicity and polarity. HOS and HRDOM have inorganic minerals which can influence adsorption. Therefore, although HOS and HRDOM have lower aromaticity and higher polarity than HRM, HRLF, and HNHC fractions, they still show a good adsorption capacity.

**Figure 12:**
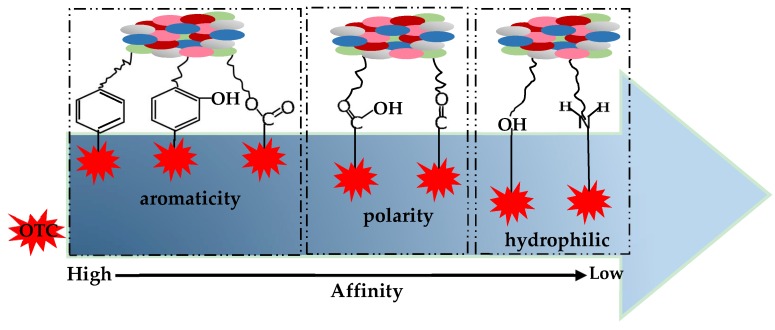
The affinity of functional groups combined with OTC.

A schematic diagram for the binding affinity of aromatic, polar, and hydrophilic functional groups to OTC is established according to infrared spectra and elemental analysis (Figure 12). Using the sequential order rules, the sequence of functional groups in the interaction process of organic matter with OTC is in the order of the aromatic functional groups, i.e., C=C, phenolic and aliphatic > the polarity groups, i.e., carboxyl and carbonyl > the hydrophilic functional groups, i.e., hydroxyl and amino.

This study is significant to control antibiotics migration to groundwater. However, due to pollutants in the environment such as antibiotics, heavy metals, and hydrophobic organic pollutants, it is necessary to further research composite pollutants adsorption feature by different fractions.

## 4. Conclusions

This work identifies the relationship between humus soil organic matter and adsorption feature of OTC. The HNHC fraction has the largest adsorption capacity due to containing more aromatic functional groups than other fractions, and controls the fate of antibiotics in the soil environment. Therefore, it is one of the most important factors that must be paid attention to when studying the fate of antibiotics in soil. The following conclusions are drawn:

1. Sorting of adsorption capacity for each fraction as: HNHC> HOS> HRDOM> HRLF> HRM. This rule is verified by the theory of site energy distribution.
2. After removing the endogenous dissolved organic matter, the adsorption capacity of humus soil is decreased while sedimentary soil is increased. This is due to DOM in humus soil containing a large amount of humic-like substances, while the DOM in sedimentary soil contains a large amount of protein-like substances.
3. For the fraction based organic matter (HRM, HRLF and HNHC), the adsorption capacity has a positive correlation with aromaticity, while it has a negative correlation with polarity and hydrophilicity. The OTC affinity of corresponding functional groups is in the order of aromatic> polarity> hydrophilic.

## Figures and Tables

**Table 1:** TOC and pH of different fractions.

Sample	HOS	HRDOM	HRM	HRLF	HNHC
TOC (%)	28.956	24.060	47.425	48.389	49.321
pH	5.86	6.91	7.03	6.81	7.02

**Table 2:** The equations of adsorption models of kinetics and isotherm.

Model	Equation	Parameters
Pseudo-first-order kinetic	$Q_{t}=Q_{e}\left(1-e^{-K_{1}t}\right)$	*Q_t_* = quantity of OTC adsorbed at any time t (mg/kg) *Q_e_* = quantity of OTC adsorbed at equilibrium (mg/kg)
pseudo-second-order kinetic	$Q_{t}=\frac{K_{2}Q_{e}^{2}t}{1+K_{2}Q_{e}t}$	*k*_1_ = pseudo-first-order rate constant (1/h) *k*_2_ = pseudo-second-order rate constant (kg/mg h)
Langmuir adsorption isotherm	$Q_{e}=\frac{Q_{max}K_{L}C_{e}}{1+K_{L}C_{e}}$	*C_e_* = OTC concentration of solution at equilibrium (mg/L) *Q_max_* = maximum adsorbed capacity (mg/kg) *K_L_* = Langmuir adsorption isotherm constant (L/mg)
Freundlich adsorption isotherm	$Q_{e}=K_{F}C_{e}^{\frac{1}{n}}$	*k_F_* = Freundlich adsorption coefficient ((mg/kg) (L/mg)^1/n^) 1/*n* = Freundlich index

**Table 3:** The adsorption kinetics model fitted parameters.

Samples	Qe,exp (mg/kg)	Pseudo-First-Order Model				Pseudo-Second-Order Model			
		R^2^	K_1_ (1/h)	Qe,cal (mg/kg)	RSS/dof	R^2^	K_2_ (kg/mg·h)	Qe,cal (mg/kg)	RSS/dof
HOS	842.10	0.8182	0.4020	807.58	381.18	0.9648	0.0007	872.29	73.73
HRDOM	732.78	0.8876	0.2697	704.68	281.80	0.9748	0.0005	779.05	63.09
HRM	553.14	0.8566	0.6527	536.78	51.52	0.9909	0.0022	561.32	3.26
HRLF	587.04	0.8817	0.6360	570.89	50.25	0.9952	0.0020	597.27	2.02
HNHC	849.83	0.7613	0.9189	821.57	78.81	0.9575	0.0027	844.86	14.03

**Table 4:** The fitted parameters for Langmuir isotherm model and Freundlich isotherm model.

Samples	Langmuir model				Freundlich Model			
	Q_m_ (mg/kg)	K_L_ (L/mg)	AIC_c_	RSS/dof	K_F_ (mg/kg) (L/mg)	1/n	AIC_c_	RSS/dof
HOS	2697.61	0.5906	34.86	721.41	1061.04	0.4469	31.46	365.88
HRDOM	2576.05	0.2231	34.28	642.38	600.81	0.4947	31.31	355.11
HRM	1687.53	0.0918	26.00	122.61	218.91	0.6364	25.02	100.83
HRLF	2377.73	0.1062	27.83	176.73	263.77	0.5940	25.99	122.54
HNHC	2737.76	0.6032	30.58	306.79	1083.64	0.3798	30.23	285.59

**Table 5:** The parameters of adsorption thermodynamics.

Samples	T (°C)	*lnK*_0_	Δ*G* (KJ/mol)	Δ*H* (KJ/mol)	Δ*S* (KJ/mol)
HOS	20	5.26	−12.81	91.86	0.3572
	30	6.49	−16.90		
	40	7.67	−19.33		
HRDOM	20	4.84	−11.80	12.98	0.0871
	30	5.94	−14.96		
	40	6.16	−16.03		
HRM	20	3.97	−9.67	36.40	0.1578
	30	4.68	−11.79		
	40	4.92	−12.80		
HRLF	20	4.46	−10.86	30.61	0.1419
	30	5.01	−12.62		
	40	5.26	−13.69		
HNHC	20	5.96	−14.52	99.26	0.3863
	30	6.57	−16.55		
	40	8.58	−22.33		

**Table 6:** Comparison of the OTC adsorption by different soil organic matter.

Extraction Treatment of Sample	Qe, exp (mg/kg)		Freundlich Model Parameter (1/n)		Thermodynamics Parameter (Δ*G*, 40 °C)	
	Sediments Soil	Humus Soil	Sediments Soil	Humus Soil	Sediments Soil	Humus Soil
Original soil	111.50	842.10	0.6199	0.4469	−8.48	−19.33
Removal of DOM	238.07	732.78	0.8386	0.4947	−10.53	−16.03
Removal of minerals	60.28	553.14	0.7716	0.6364	−5.16	−12.80
Removal of free fat	100.8	587.04	0.7338	0.5940	−9.06	−13.69
Removal of sugar and protein	219.15	849.83	0.8569	0.3798	−10.93	−22.33

**Table 7:** Physical properties of different fractions.

sample	HOS	HRDOM	HRM	HRLF	HNHC
Surface Area m^2^/g	−0.1983 ± 0.2634	0.5712 ± 0.8463	5.2148 ± 3.2002	137.5180 ± 125.9391	−0.3817 ± 0.5365
micropore volume cm^3^/g	0.007275	\	\	\	0.002181

**Table 8:** Elemental composition of different humus fractions.

Samples	C%	H%	N%	O%	TOC (%)	H/C	O/C	(N+O)/C	K_d_
HOS	26.05	3.19	1.95	30.81	28.96	1.47	0.89	0.95	381.20
HRDOM	27.03	3.40	1.73	32.39	24.06	1.51	0.90	0.95	194.48
HRM	47.06	4.70	2.92	39.72	47.43	1.20	0.63	0.69	67.05
HRLF	47.78	4.81	3.01	41.08	48.39	1.19	0.63	0.68	93.36
HNHC	49.23	4.24	1.75	38.18	49.32	1.03	0.58	0.61	425.59

## Abbreviations

OTC	Oxytetracycline
Phen	Phenanthrene
HOS	Humus soil
HRDOM	Removal of dissolved organic matter
HRM	Removal of minerals
HRLF	Removal of free fat
HNHC	nonhydrolyzable organic carbon
FA	Fulvic acid
HA	Humic acid
HM	Humin
KC	kerogen graphitic
BC	black carbon
TOC	The total organic carbon
UPLC	ultra-performance liquid chromatography
PDOM	Dissolved organic matter from decay plant
MDOM	Dissolved organic matter from chicken manure
